# A rare coexistence of hypoplasia of the middle cerebral artery with aortic arch origin of the vertebral artery: a case report and analysis of hemodynamic vulnerability

**DOI:** 10.1186/s12883-026-04775-y

**Published:** 2026-02-28

**Authors:** Jiazhen Li, Yinbao Hu

**Affiliations:** Department of Neurology, Yantai Yuhuangding Hospital Affiliated with Qingdao University, NO. 20 East Yuhuangding Road, Yantai, Shandong 264000 China

**Keywords:** Middle cerebral artery hypoplasia, Vertebral artery origin anomaly, Hemodynamic insufficiency, Circle of Willis, Refractory dizziness

## Abstract

**Background:**

Congenital cerebrovascular anomalies, such as middle cerebral artery hypoplasia and vertebral artery with an aortic arch origin, are well-documented in isolation but exceedingly rare in combination. Their coexistence may critically disrupt cerebral hemodynamics, yet clinical implications remain poorly characterized, highlighting the novelty of this case.

**Case presentation:**

A 68-year-old male presented with chronic refractory dizziness that worsened with positional changes and showed no improvement after standard management for benign paroxysmal positional vertigo. Comprehensive neurovascular imaging, including multimodal angiography, revealed two coexisting anomalies—left middle cerebral artery hypoplasia with duplicate trunks and distal tortuosity, along with an aberrant left vertebral artery originating directly from the aortic arch, which exhibited extracranial tortuosity with impaired flow and was identified as non-dominant. The hemodynamic vulnerability was further compounded by a right fetal-type posterior cerebral artery and a patent left posterior communicating artery, with an anterior communicating artery that was not visualized. The patient received antiplatelet and statin therapy, but the symptoms persisted, underscoring the refractory nature of the condition.

**Conclusions:**

This unique constellation of anomalies may create a fragile hemodynamic state, which likely contributed to the patient’s refractory symptoms. This case underscores the importance of comprehensive vascular evaluation, including assessment of the circle of Willis, in patients with unexplained neurological symptoms. Advanced angiographic assessment is crucial for accurate diagnosis and to avoid misinterpreting congenital variants as acquired pathology.

## Background

The middle cerebral artery (MCA) is a critical vessel for cerebral blood supply, and its congenital variations, though rare, can significantly alter cerebral hemodynamics [1]. MCA hypoplasia encompasses a spectrum of anomalies, including moyamoya syndrome (characterized by progressive steno-occlusive changes and collateral network formation), reticulated MCA (a fenestrated or plexiform arterial pattern), and duplicate MCA (accessory vessels with hypoplastic features) [2, 3]. These variants are often asymptomatic individually but may predispose to hemodynamic insufficiency or be misdiagnosed as acquired stenosis [3, 4]. Concurrently, an aberrant origin of the vertebral artery (VA)—most commonly directly from the aortic arch—is observed in 0.5%–6% of the population, potentially exacerbating posterior circulation compensatory limitations [5]. Critically, the functional integrity of the circle of Willis plays a pivotal role in mitigating hemodynamic impairment caused by such variations [6]. An incomplete or variant Willisian configuration, frequently observed in conjunction with proximal cerebrovascular anomalies, can severely limit collateral flow compensation, further elevating the risk of cerebral hypoperfusion, particularly under physiological stress or postural changes [7]. While both entities are well-documented in isolation, their coexistence remains exceedingly rare, and no systematic reports have described their combined hemodynamic and clinical implications.

Embryologically, these anomalies may stem from disruptions during the 7th–8th gestational week. MCA develops from a plexiform network of arteries. MCA hypoplasia may therefore originate from the failed regression of this early embryonic arterial plexus or from anomalous budding of the internal carotid artery [8]. Similarly, an aortic arch origin of the left vertebral artery (VA) is thought to result from the persistence of a segment derived from the dorsal aorta, coupled with a failure to establish a normal connection to the subclavian artery [5]. This shared developmental window suggests a potential, though not proven, linked mechanism, although their concurrent presentation could also be coincidental. Hemodynamically, the interplay between a hypoplastic anterior circulation and a compromised posterior circulation creates a vulnerable state characterized by critically diminished collateral reserves. The significance of this report lies not merely in the anatomical rarity of this coexistence, but in the cumulative hemodynamic impact of these variants within the context of an incomplete circle of Willis.

Given the scarcity of reported cases and the potential for clinical sequelae, this case report aims to: (1) elucidate the imaging characteristics of coexisting MCA hypoplasia and aberrant VA origin using multimodal angiography; (2) analyze the resultant hemodynamic compromise as a likely contributing factor to the clinical symptoms, with particular emphasis on the role of an incomplete circle of Willis; and (3) emphasize the necessity of comprehensive vascular evaluation in patients with unexplained neurological deficits to avoid diagnostic pitfalls. Our findings seek to enhance clinical recognition of this rare constellation and inform long-term management strategies.

### Case presentation

A 68-year-old male presented with a 3-year history of progressive dizziness, exacerbated by positional changes (e.g., turning in bed, looking up) and accompanied by gait instability, lower limb weakness, and autonomic symptoms (e.g., diaphoresis and palpitations). A prior diagnosis of benign paroxysmal positional vertigo (BPPV) and subsequent canalith repositioning maneuvers provided only minimal relief. His past medical history was significant for reflux esophagitis, gastric ulcer, and an anxiety disorder. His vascular risk factors included a 40-pack-year smoking history (with cessation three years prior). A comprehensive neurological examination was unremarkable, revealing no focal deficits, cranial nerve abnormalities, or signs of long-tract dysfunction.

### Imaging findings

**Fig. 1:**
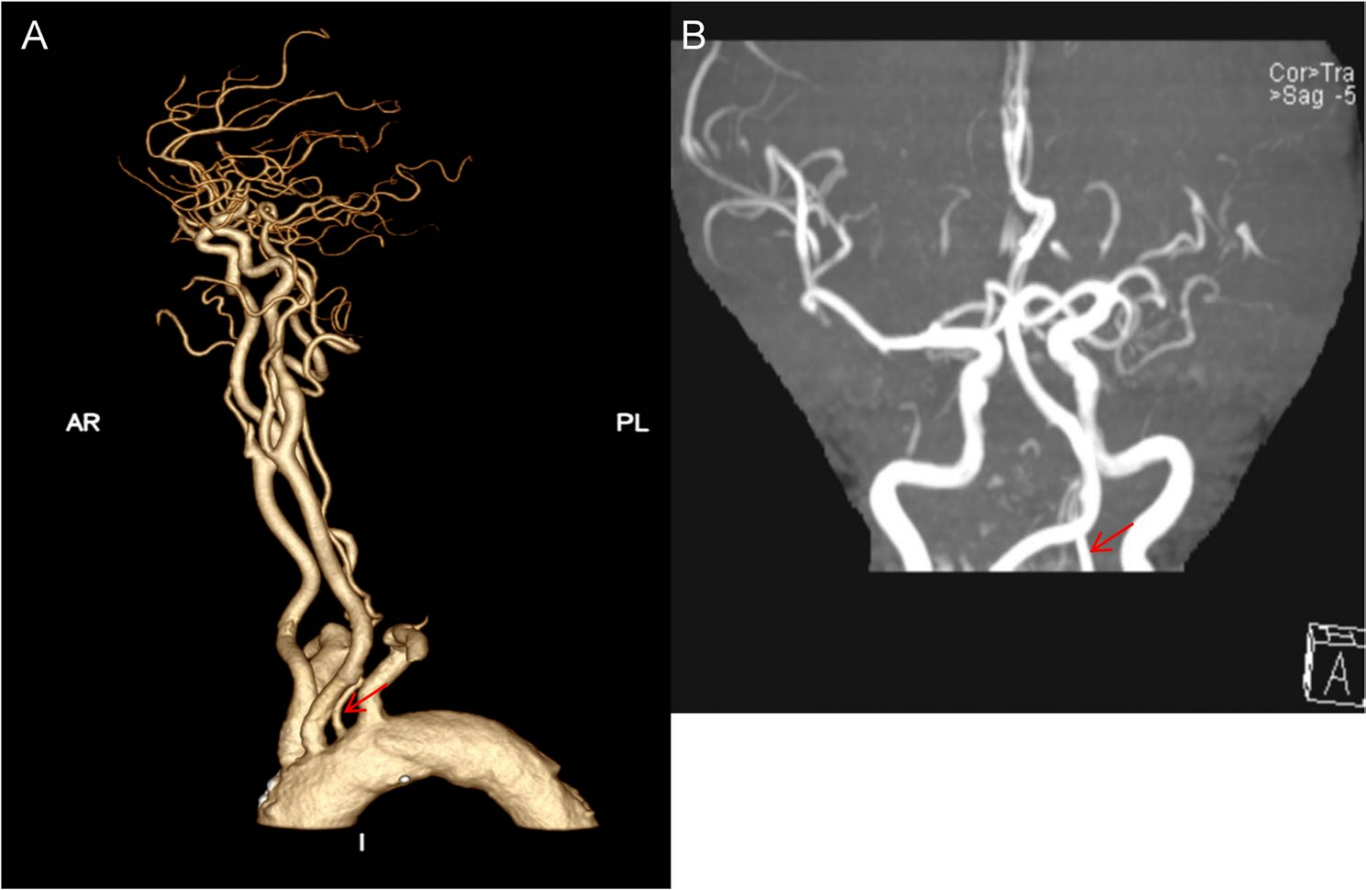
Aberrant origin of the left vertebral artery (LVA) from the aortic arch with hypoplastic and non-dominant features. **A** Illustration depicting the LVA (red arrow) originating directly from the aortic arch. **B** Angiographic image (MRA) showing a hypoplastic, non-dominant LVA (red arrow) associated with this variant

Comprehensive vascular imaging delineated the patient’s complex cerebrovascular anatomy. Computed Tomography Angiography (CTA) (Fig. 1A) revealed the left VA arising directly from the aortic arch, with its origin situated between the left common carotid and subclavian arteries. Magnetic Resonance Angiography (MRA) (July 29, 2025) further demonstrated left MCA hypoplasia with duplicate trunks and distal tortuosity. The hypoplasia was defined by a visibly diminished caliber of the left MCA main trunk, estimated to be less than 50% of the diameter of the contralateral right MCA on axial MRA images, in the absence of focal stenosis or signs of acquired disease. This finding was accompanied by notable vertebrobasilar tortuosity; on this study, the left VA was also specifically noted to be non-dominant **(**Fig. 1B).

**Fig. 2:**
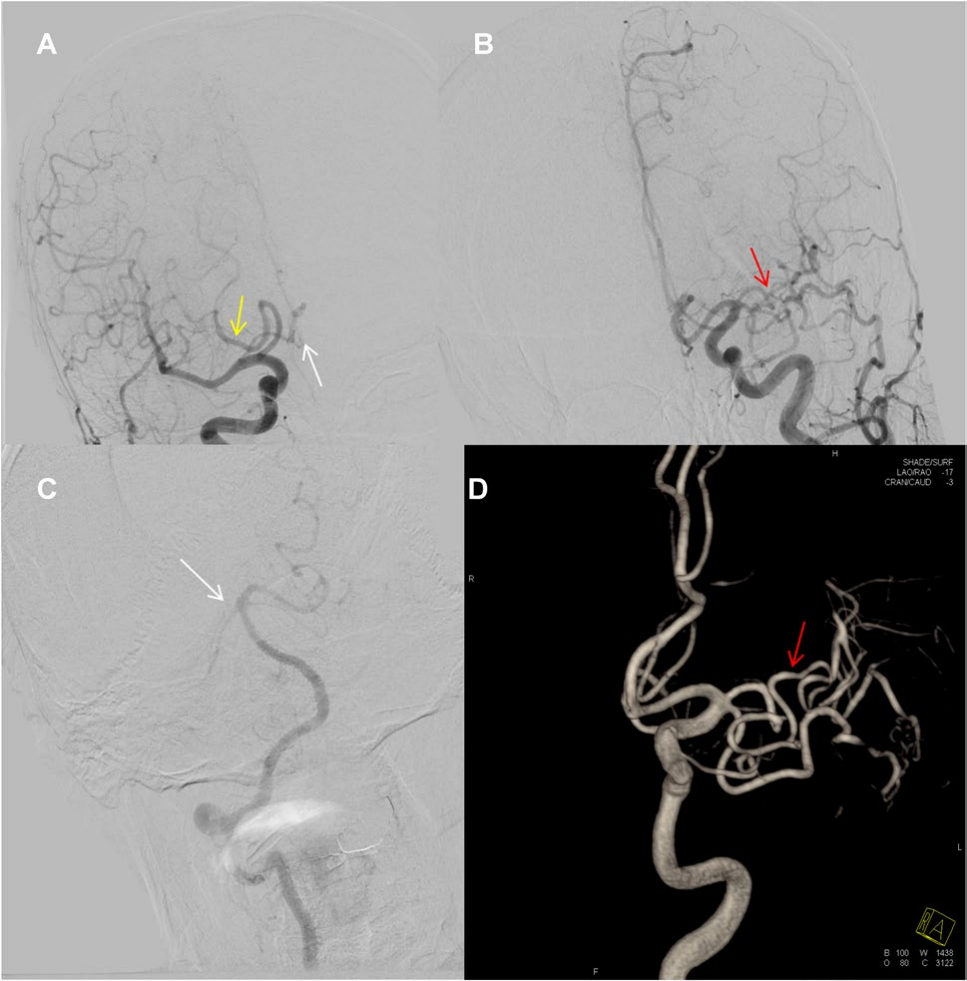
Cerebral angiography demonstrating the incomplete circle of Willis and MCA hypoplasia. **A** Right internal carotid artery (ICA) angiography, anteroposterior view, shows a fetal-type posterior cerebral artery (fPCA, yellow arrow), where the PCA is predominantly supplied by the ICA via a prominent posterior communicating artery (Pcom). The anterior communicating artery (ACom) is poorly visualized (white arrowhead). **C** Right vertebral artery angiography, lateral view, confirms the fetal-type PCA by demonstrating non-visualization of the right PCA (white arrowhead) from the vertebrobasilar system. **B**,** D** Left common carotid artery angiography, anteroposterior view (**B**) and 3D volume-rendered reconstruction (**D**), showing a duplicate left middle cerebral artery (MCA) with a hypoplastic main trunk (red arrows) and an accessory trunk. Distal tortuosity is also evident

Digital Subtraction Angiography (DSA) confirmed the CTA and MRA findings, definitively ruling out acquired stenosis or occlusion. It confirmed a duplicate left MCA with a hypoplastic trunk and distal tortuosity (Fig. 2B and D). Furthermore, it identified a right fetal-type posterior cerebral artery (PCA) and a patent left posterior communicating artery (Pcom). The anterior communicating artery (ACom) was poorly visualized on DSA, suggesting it is either hypoplastic or associated with slow flow, rendering it functionally insignificant, although its patency cannot be definitively ruled out without specific flow studies (Fig. 2A and C). Laboratory investigations, including inflammatory markers and a thrombophilia screen, were unremarkable. A transthoracic echocardiogram and ECG showed no evidence of a cardioembolic source.

### Management and outcome

The patient was managed with antiplatelet therapy (aspirin 100 mg daily), a high-intensity statin (atorvastatin 40 mg daily), and specific microcirculatory improvement agents. Anxiolytics were continued for his pre-existing anxiety disorder. Although symptomatic improvement was minimal, he was discharged in stable condition. At a six-month follow-up clinic visit, the patient reported that his dizziness and gait instability were stable but persistent, with no new neurological events. He remains on the same medical therapy. Annual clinical and neurovascular imaging (MRA) follow-up has been recommended to monitor for disease progression or the development of flow-related aneurysms.

## Discussion and conclusions

This case represents a notable confluence of rare cerebrovascular anomalies that collectively create a fragile hemodynamic state. While their coexistence could be coincidental, the cumulative impact of left MCA hypoplasia and an aberrant, non-dominant left VA origin, as documented in our patient, is likely the anatomical substrate for a critical reduction in hemodynamic reserve. This vulnerability was exacerbated by an incomplete circle of Willis. The hypoplastic MCA, with its duplicate trunk and attenuated flow, reduces perfusion reserve in the left hemisphere. Critically, the left VA was non-dominant. While an anomalous origin of a non-dominant VA is often asymptomatic in isolation, in this patient its tortuous course and impaired distal flow compromise its potential role as a collateral pathway to the posterior circulation. This places a greater hemodynamic burden on the dominant right VA and the circle of Willis, particularly during physiological stressors like head movement. This interpretation aligns with studies showing that anomalous VAs are more frequently hypoplastic and may have altered flow dynamics [9–11].

A central factor in this hemodynamic compromise is the incomplete circle of Willis, which has been emphasized as pivotal in mitigating impairment induced by vascular variations [12]. DSA revealed a right fetal-type PCA and patent left Pcom. However, the anterior communicating artery (ACom) was not visualized, indicating a variant configuration that provides only partial collateral efficacy. This incomplete network fails to facilitate robust cross-hemispheric or anterior-posterior compensation. The absence of a functional ACom, in particular, restricts anterior collateralization, exacerbating the vulnerability created by the MCA hypoplasia [13]. This observation aligns with prior research highlighting how Willisian anomalies can elevate hypoperfusion risk under stress.

The patient’s clinical presentation—positionally exacerbated dizziness, gait instability, and autonomic symptoms—raises a differential diagnosis that includes BPPV, central vertigo, and anxiety-related dizziness. However, the refractoriness to BPPV treatment and the absence of focal neurological signs make a hemodynamic etiology plausible. We hypothesize that his symptoms represent transient hypoperfusion in watershed zones, potentially mimicking transient ischemic attacks (TIAs). Head movement likely worsens flow through the tortuous VA while simultaneously straining the inefficient Willisian collaterals. The lack of perfusion imaging to confirm this mechanism is a limitation, but the anatomical findings provide the most compelling explanation for the symptoms’ chronic and refractory nature.

The novelty of this case is underscored by a review of the literature. While MCA hypoplasia and aberrant VA origins have been individually well-described, their coexistence, particularly with a detailed analysis of the circle of Willis and its clinical impact, has not been reported (see Table 1). This case highlights how the cumulative effect of multiple variants can lead to symptoms where any single variant might remain silent.

**Table 1:** Summary of previously reported similar variants

Study	Variant Reported	Imaging	Presentation	Key Findings / Novelty of Our Case
Kashtiara et al. (2024) [1]	MCA anomalies (review)	Mixed	Variable	Comprehensive review, but no focus on combined anomalies.
Uchiyama (2017) [2]	MCA anomalies	Mixed	Variable	Describes duplicate/hypoplastic MCAs but not in combination with arch vessel anomalies.
Yuan (2016) [5]	Aberrant VA origin	Mixed	Often incidental	Focuses solely on vertebral artery origin, not concurrent anterior circulation variants.
Akkan et al. (2015) [3]	Twig-like MCA	DSA	Stroke/TIA	Discusses hemodynamic impact of MCA anomaly alone.
Kim et al. (2017) [10]	Anomalous VA and hypoplasia	CTA/MRA	Asymptomatic	Correlates anomalous origin with hypoplasia but not with symptoms or complex Willisian variants.
**Present Case**	**Coexisting MCA hypoplasia + aberrant VA origin + incomplete Willis**	**CTA**,** MRA**,** DSA**	**Refractory positional dizziness**	**First to describe the cumulative hemodynamic effect of this triad and its correlation with refractory symptoms.**

This case highlights several critical clinical implications. First, unexplained and treatment-resistant dizziness necessitates a comprehensive vascular workup including detailed assessment of the circle of Willis. Although MRA and CTA serve as valuable screening modalities, DSA remains indispensable for delineating complex variants and ruling out acquired pathologies like atherosclerosis, dissection, or vasculitis. Second, recognizing these variants has practical implications for acute stroke triage and intervention: a hypoplastic MCA could be mistaken for an occlusion, and an aberrant VA origin is critical information for planning thrombectomy, as it may alter arch navigation and catheter selection. Third, intensive medical management, including antiplatelet and statin therapy, is prudent to mitigate thromboembolic risks in low-flow states. Finally, patients require regular monitoring for both ischemic and hemorrhagic complications, as altered flow dynamics may predispose to hypoperfusion-related ischemia and flow-induced aneurysms.

This study has several limitations. As a retrospective case report, it is inherently descriptive. Most importantly, advanced perfusion imaging (CTP or MRP) was not performed, which prevents us from quantifying the hemodynamic significance of the anomalies and definitively linking them to the patient’s symptoms. The lack of functional assessment of the ACom (e.g., with a Matas test during angiography) also limits our ability to fully characterize collateral capacity. Furthermore, while we hypothesize a hemodynamic basis for the positional symptoms, we lack direct imaging evidence of flow changes during head movement.

## Conclusions

In summary, the concurrence of left MCA hypoplasia and an aberrant, non-dominant VA origin represents a rare anatomical constellation. The incomplete circle of Willis—demonstrated by a fetal-type PCA and an unvisualized ACom—likely exacerbates this compromise by limiting collateral reserve, contributing to the patient’s refractory positional symptoms. This case reinforces the necessity of comprehensive vascular evaluation, including detailed assessment of the circle of Willis, to enable precise diagnosis, avoid mislabeling congenital variants as acquired disease, and tailor management. Enhanced clinician awareness of these interactions can improve outcomes through early and appropriate intervention. Future studies should focus on advanced hemodynamic mapping to optimize strategies for such complex variations.

## Data Availability

The datasets generated during and/or analysed during the current study are not publicly available due to ethical and privacy restrictions (to protect patient anonymity) but are available from the corresponding author on reasonable request.

## Abbreviations

ACom: Anterior Communicating Artery
BPPV: Benign Paroxysmal Positional Vertigo
CTA: Computed Tomography Angiography
CTP: Computed Tomography Perfusion
DSA: Digital Subtraction Angiography
ECG: Electrocardiogram
fPCA: Fetal-type Posterior Cerebral Artery
ICA: Internal Carotid Artery
MCA: Middle Cerebral Artery
MRA: Magnetic Resonance Angiography
MRP: Magnetic Resonance Perfusion
NIHSS: National Institutes of Health Stroke Scale
PCA: Posterior Cerebral Artery
Pcom: Posterior Communicating Artery
SPECT: Single Photon Emission Computed Tomography
TIA: Transient Ischemic Attack
TTE: Transthoracic Echocardiogram
VA: Vertebral Artery
